# Fucoidan and Fucosylated Chondroitin Sulfate Stimulate Hematopoiesis in Cyclophosphamide-Induced Mice

**DOI:** 10.3390/md15100301

**Published:** 2017-09-30

**Authors:** Natalia Anisimova, Nadezhda Ustyuzhanina, Maria Bilan, Fedor Donenko, Anatolii Usov, Mikhail Kiselevskiy, Nikolay Nifantiev

**Affiliations:** 1 N.N. Blokhin Medical Research Center of Oncology, Ministry of Health of the Russian Federation, Kashirskoe Shosse, 24, 115478 Moscow, Russia; n_anisimova@list.ru (N.A.); donenko.f20010@yandex.ru (F.D.); 2 N.D. Zelinsky Institute of Organic Chemistry, Russian Academy of Sciences, Leninsky Prospect 47, 119991 Moscow, Russia; ustnad@gmail.com (N.U.); bilan@ioc.ac.ru (M.B.); usov@ioc.ac.ru (A.U.)

**Keywords:** granulocyte colony-stimulating factor, fucoidan, fucosylated chondroitin sulfate, hematopoiesis, immunosuppression, cyclophosphamide

## Abstract

Application of cytostatics in cancer patients’ chemotherapy results in a number of side effects, including the inhibition of various parts of hematopoiesis. Two sulfated polysaccharides, fucoidan from the seaweed *Chordaria flagelliformis* (**PS-Fuc**) and fucosylated chondroitin sulfate from the sea cucumber *Massinium magnum* (**PS-FCS**), were studied as stimulators of hematopoiesis after cyclophosphamide immunosuppression in mice. Recombinant granulocyte colony-stimulating factor (**r G-CSF**) was applied as a reference. Both tested polysaccharides **PS-Fuc** and **PS-FCS** have a similar activity to **r G-CSF**, causing pronounced neutropoiesis stimulation in animals with myelosuppression induced by cyclophosphamide (**CPh**). Moreover, these compounds are also capable to enhance thrombopoiesis and erythropoiesis. It should be noted that **PS-FCS** demonstrated a greater activity than **r G-CSF**. The results indicate the perspective of further studies of **PS-Fuc** and **PS-FCS**, since these compounds can be considered as potentially promising stimulators of hematopoiesis. Such drugs are in demand for the accompanying treatment of cancer patients who suffer from hematological toxicity during chemo and/or radiation therapy.

## 1. Introduction

Chemotherapy of cancer patients with cytostatics results in a number of side effects, including hematopoietic toxicity. One of the widely applied chemotherapy drugs with a large spectrum of antitumor activity is cyclophosphamide (**CPh**). It is an alkylating agent used in the treatment of various forms of malignant neoplasms [1].

The main toxic effect of **CPh** is an acute inhibition of hematopoiesis, which manifests by the suppression of rapidly proliferating hematopoietic progenitor cells and results in the form of neutropenia. However, lymphopenia and erythropenia are also significant toxic side effects of **CPh** [2,3]. In clinical practice, cancer patients’ treatment with **CPh** and other cytotoxic agents is accompanied by marked thrombocytopenia, which was manifested by the inhibitory effect of **CPh** on later megakaryocyte progenitors [4].

At present, recombinant granulocyte colony-stimulating factor (**r G-CSF**) is applied to combat the most severe complication of chemotherapy-induced neutropenia. Currently, there are various **r G-CSF** dosage forms that are used to treat neutropenia caused by chemo- and/or radiotherapy and to stimulate the recovery of neutrophils after bone marrow transplantation in cancer patients [5]. For preclinical evaluation of neutropoiesis stimulation efficiency under the action of **r G-CSF**, small laboratory animals (mice and rats) with induced myelosuppression are used [6]. Experimental studies in mice with induced myelosuppression have shown that **r G-CSF** stimulates not only the colony formation of bone marrow precursor cells but also causes marked stimulation of hematopoiesis in the spleen [7]. However, **r G-CSF** drugs do not have a stimulating effect on platelet and erythrocyte germ. Therefore, in patients with combined neutro-thrombocytopenia and erythrocytopenia, additional stimulation of thrombopoietin (erythropoietin) factors or transfusion of blood components (platelets, erythrocytes) are demanded.

Various compounds of polysaccharide nature were found to stimulate hematopoiesis similarly to colony-stimulating factor [8,9,10]. At the same time, in experimental models it was shown that, unlike **r G-CSF**, some of these compounds on the background of cyclophosphamide-induced myelosuppression not only neutralize neutropenia, but also contribute to the restoration of the number of lymphocytes and red blood cells (RBC) in animals. In particular, sulfated polysaccharide fucoidan from the seaweed *Fucus vesiculosis* caused pronounced mobilization of progenitor cells when administered to intact mice at a dose of 25 mg/kg. The colony-stimulating effect of the fucoidan was associated with its ability to inhibit P-selectin and L-selectin but not E-selectin [8,11]. 

Recently, various biological properties similar to those of fucoidan were shown for another type of sulfated polysaccharides, namely, fucosylated chondroitin sulfates [12,13,14,15]. These compounds from different species of sea cucumbers vary in structure, including degree and pattern of sulfation, position of branches and molecular weight [14,15,16,17]. It is known that the fine structure of O-sulfated polysaccharides significantly influences their biological properties [17,18,19].

**Figure 1:**
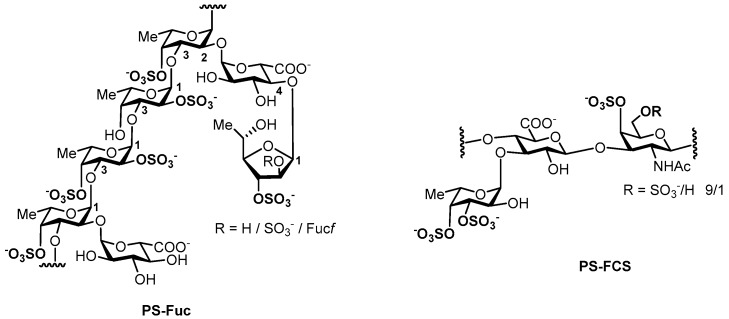
A dominant structural fragment of fucoidan (**PS-Fuc**) from the seaweed *Chordaria flagelliformis* [20] and the repeating unit of fucosylated chondroitin sulfate (**PS-FCS**) from the sea cucumber *Massinium magnum* [14].

In this communication, the results of the study of two sulfated polysaccharides as stimulators of hematopoiesis are presented. The tested compounds were fucoidan from the seaweed *Chordaria flagelliformis* (**PS-Fuc**) [20] and fucosylated chondroitin sulfate from the sea cucumber *Massinium magnum* (**PS-FCS**) [14] (Figure 1). Both polysaccharides were structurally characterized recently using chemical and physicochemical methods including comparison of their NMR spectra with those of synthetic related oligosaccharides [20,21,22,23,24,25]. **PS-FCS** represents one of the most regular and structurally simple cases of biopolymers of this group while fucoidan **PS-Fuc** was selected for its unusually complex structure.

## 2. Results

Both compounds **PS-Fuc** and **PS-FCS** are related to sulfated polysaccharides of marine origin. However, the fine structures of these biopolymers are quite different (Figure 1). The backbone of fucoidan **PS-Fuc** is built from (1→3)-linked sulfated α-l-fucopyranosyl residues, some of which bear α-d-GlcA or more complicated sulfated α-l-Fuc*f*(1→4)-α-d-GlcA fragments as branches at O-2. Polysaccharide **PS-FCS** consists of the chondroitin core [→4)-β-d-GlcA-(1→3)-β-d-GalNAc-(1→]_n_ decorated by 3,4-di-O-sulfated α-l-fucosyl branches attached to O-3 of GlcA units and sulfates at O-4 and/or O-6 of GalNAc.

**Figure 2:**
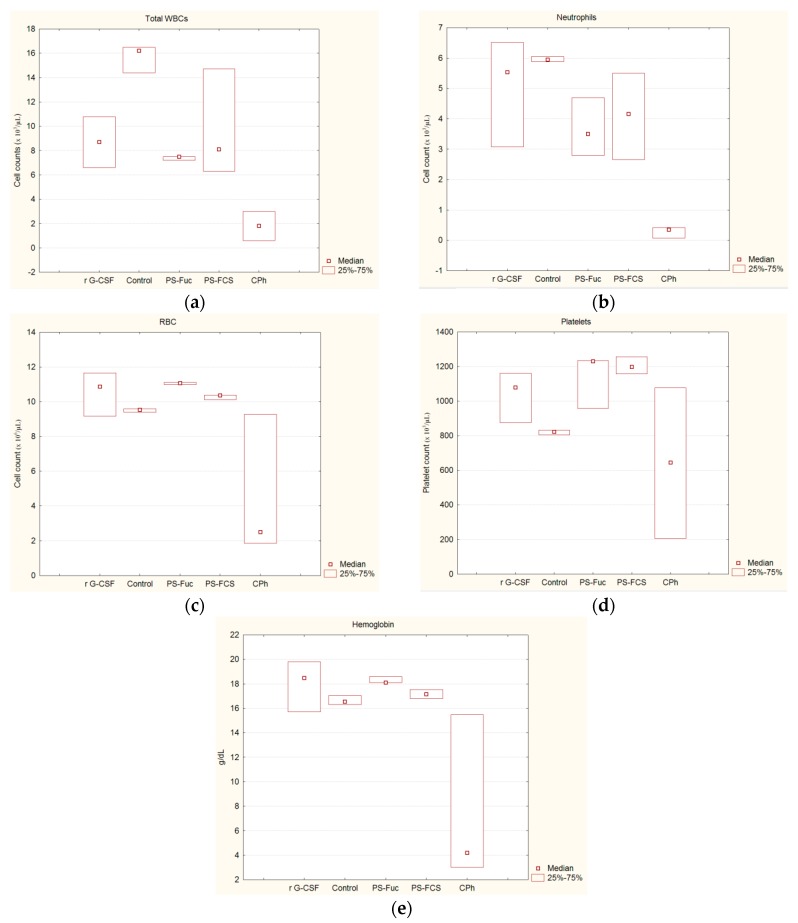
Hematologic parameters of mice with **CPh**-induced immunosuppression after the treatment with **PS-Fuc**, **PS-FCS** and **r G-CSF**: (**a**) WBC; (**b**) neutrophils; (**c**) red blood cells (RBC); (**d**) platelets; (**e**) hemoglobin. Intact animals were characterized as a control.

The study of the influence of polysaccharides **PS-Fuc** and **PS-FCS** on hematopoiesis was performed on the model of **CPh**-induced immunosuppression in mice. Recombinant **r G-CSF** (Leicyta) was applied as a reference. Intact animals were characterized as a control. Used active concentrations were selected on the basis of our previous data [18,26]. The results of the investigation of hematological parameters in various groups of mice are presented in Table 1 and Figure 2. The levels of white blood cells (WBC), neutrophils, RBC, platelets and hemoglobin were determined.

The data of Table 1 indicated that under the influence of **PS-Fuc**, **PS-FCS** and **r G-CSF** there was a significant (*p* ≤ 0.041) increase of WBC in comparison with the group **CPh**: 4.2, 4.5 and 4.8 times, respectively (by the ratio of medians). This indicated the leveling of **CPh**-induced leukopenia after the administration of **PS-Fuc** and **PS-FCS**. At the same time, the level of their activity was not significantly different from that of **r G-CSF** (*p ≥* 0.345). The recovery of total WBC count under the influence of both tested compounds was due to an increase in the neutrophils count in the blood of mice with **CPh**-induced leukopenia (*p* ≤ 0.01). This trend was more pronounced under the influence of **PS-FCS** and **r G-CSF** than under the influence of **PS-Fuc**: the neutrophils count increased by 10.5, 13.8 and 8.8 times, respectively, compared with the group of **CPh**.

An increase of the concentration of RBC, hemoglobin and platelets in the blood of mice with **CPh**-induced immunosuppression was observed after the treatment with **r G-CSF**, **PS-Fuc** and **PS-FCS**. It was noticeable that in the case of the use of **PS-Fuc** and **PS-FCS** increasing of RBC, hemoglobin and platelets was statistically significant (*p* < 0.05) comparing to a control, while in the case of **r G-CSF** the effect was not significant because the *p* values remarkably exceeded 0.05 (0.226 for RBC, 0.328 for hemoglobin, and 0.063 for platelets, Table S1).

**Figure 3:**
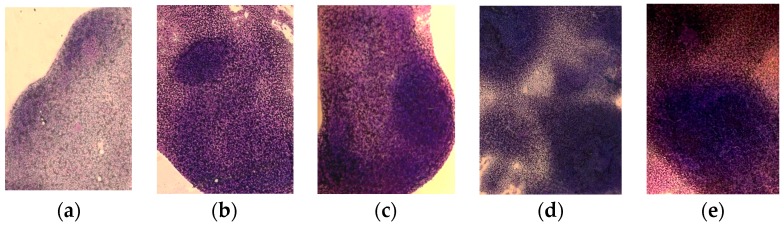
Morphology of the spleen of mice with **CPh**-induced immunosuppression after treatment by tested substances compared to intact animals (hematoxylin-eosin staining): (**a**) **CPh**; (**b**) **CPh** + **PS-Fuc**; (**c**) **CPh** + **PS-FCS**; (**d**) **CPh** + **r G-CSF**; (**e**) control. Original magnification × 400.

Analysis of the morphology of the spleen on the smears-prints showed that after repeated administration of **CPh**, myelosuppression is accompanied by depletion of the cellular composition of the white pulp, represented mainly by WBC (Figure 3a). Thus, the follicles (B-dependent areas) and periarteriolar sheaths (T-dependent areas) disappeared. The architecture of the spleen was destroyed, and the interstitial tissue was composed of a dense and uniform layer of lymphoid cells. After the course of **r G-CSF** and both tested polysaccharides **PS-Fuc** and **PS-FCS**, cell repair was observed (Figure 3b–d). In particular, intensive recovery was noted after the course of **PS-FCS** administration.

## 3. Discussion

It was found that polysaccharides **PS-Fuc** and **PS-FCS**, similarly to **r G-CSF**, are able of stimulating neutropoiesis in systemic administration to mice with myelosuppression induced by **CPh**. At the same time, only **PS-FCS** in some animals led to a complete recovery of the level of total WBC to the initial values. Therefore, **PS-FCS** is more active than **PS-Fuc** in neutralization of **CPh**-induced neutropenia.

The investigated compounds also stimulated lymphopoiesis. This is evidenced by the normalization of the spleen white pulp cell composition in **CPh**-induced mice.

Concerning the effect of tested compounds on erythropoiesis, the following can be noted: the application of all three substances led to an increase in the number of RBC in peripheral blood of animals exposed to **CPh** by more than 4 times. In this case, **PS-Fuc** and **PS-FCS** acted more reproducibly than **r G-CSF** alone, leading to an increase in the concentration of RBC in the blood of animals, even above the basis level (*p* < 0.002).

Similar dynamics were observed in the analysis of the concentration of platelets in the blood of animals. It was shown that **PS-Fuc** and **PS-FCS** were more active (by 91% and 86%, respectively), compared to **r G-CSF** (by 67%) in the stimulation of thrombopoiesis in mice on the background of immunosuppression induced by **CPh**. This resulted in a significant increase in the platelet count in the blood after the administration of the test compounds in comparison with the baseline level (*p* = 0.025 and *p* = 0.001, respectively).

## 4. Materials and Methods

### 4.1. Sulfated Polysaccharides

Fucoidan **PS-Fuc** was isolated from the seaweed *Chordaria flagelliformis* as described [20]. Fucosylated chondroitin sulfate **PS-FCS** was isolated from the sea cucumber *Massinium magnum* as described [14].

### 4.2. Animal Model

The study was approved by the local ethical committee of the N.N. Blokhin Medical Research Center of Oncology, Russia, Moscow.

Thirty mice of the CBA line (males, weight 25 ± 1 g) were divided into five groups of six animals in each. Before and during the experiment, the animals were in standardized vivarium conditions (T of air was 20 ± 2 °C, under conditions of free access to food and water). For the inducing of myelosuppression, **CPh** (Endoxan, Baxter, Halle, Germany) in a dosage of 100 mg/kg was injected to animals of 4 groups 1 time daily intraperitoneally for 4 days. Then the following sterile solutions (0.2 mL) were administered subcutaneously to all animals for 3 days (1 time daily): 0.5 mg/mL of **PS-Fuc** in isotonic sodium chloride solution (group **CPh** + **PS-Fuc**), 0.5 mg/mL of **PS-FCS** in isotonic sodium chloride solution (group **CPh** + **PS-FCS**), and 3 nmol/mL of **r G-CSF** (Leucita, Sygardis AqVida, Lich, Germany) in isotonic sodium chloride solution (group **CPh** + **r G-CSF**), sterile isotonic sodium chloride solution (groups **CPh**). A sterile isotonic sodium chloride solution was administered to the mice of the control group in the same regime. The animals were euthanized by decapitation after 2 days. Blood of each animal was collected in the tubes with ethylenediaminetetraacetic acid (EDTA), the spleen was removed from the animals, and smears were imprinted on the polyethylene coated glasses (Gerhard Menzei GmbH, Termo Fisher Scientific, Waltham, MA, USA). The fingerprints were fixed in May-Grunwald solution, stained with hematoxylin-eosin (HE) and analyzed by light microscopy. Hematologic parameters of blood were analyzed on an automatic analyzer, determining the concentration of WBC, platelets and RBC. In a blood smear stained with HE, a neutrophil count of 100 leukocytes was counted using a light microscope, then the number of neutrophils in the blood of mice was calculated.

### 4.3. Statistical Analysis

The data in the group was represented in the format of medians and range of minimum–maximum values. The differences between multiple groups were compared with an ANOVA. The Wald-Wolfowitz runs test was used to compare the two groups. Differences were considered significant at *p* < 0.05.

## 5. Conclusions

Two sulfated polysaccharides as stimulators of hematopoiesis have been studied in vivo. The tested compounds were the fucoidan from the seaweed *C. flagelliformis* (**PS-Fuc**) and the fucosylated chondroitin sulfate from the sea cucumber *M. magnum* (**PS-FCS**). Both tested biopolymers have a comparable level of activity with **r G-CSF** with regard to stimulation of pronounced neutropoiesis in animals with myelosuppression induced by **CPh**. Additionally, these compounds were shown to be capable of stimulating thrombopoiesis and erythropoiesis. It should be noted that **PS-FCS** demonstrated a greater activity than **r G-CSF**.

The obtained results argue for the necessity of further studies into **PS-Fuc**- and **PS-FCS-**like polysaccharides and synthetic oligosaccharides which represent their fragments to form the basis for further development of novel hematopoiesis stimulating drugs. They are in demand for the accompanying treatment of cancer patients who suffer from hematological toxicity during chemo and/or radiation therapy. Availability of parent natural fucoidans [18,20,21] and fucosylated chondroitin sulfates [12,13,14,15,16,17] of very different structure as well as the possibility of chemical synthesis of oligosaccharides which represent fucoidans [22,25,26,27] and fucosylated chondroitin sulfates [23,24] fragments as well as the methods for their conformational analysis [27,28,29,30,31] permits systematic structure-activity study, to assess the pharmacophore fragments in the structures of sulfated polysaccharides and to undertake the next steps—the search for target cellular receptors and rational design of their druggable inhibitors. These studies are in progress and will be reported in due course.

## Figures and Tables

**Table 1:** Hematologic parameters of mice with cyclophosphamide (**CPh**)-induced immunosuppression after treatment with **PS-Fuc**, **PS-FCS** and recombinant granulocyte colony-stimulating factor (**r G-CSF**).

Groups	WBC (×10^3^/µL)	Neutrophils (×10^3^/µL)	Neutrophils (%)	RBC (×10^6^/µL)	Hemoglobin (g/dL)	Platelets (×10^3^/µL)
**CPh** + **r G-CSF**	8.7 ^1^	5.5	51	10.9	18.5	1079
	6.9–10.9 ^2^	3.0–6.9	45–77	9.0–11.9	14.9–19.9	870–1169
**CPh** + **PS-Fuc**	7.5 ^1^	3.5	47	11.1	18.1	1231
	7.3–7.7 ^2^	2.1–4.9	36–69	11.0–11.6	18.0–18.9	951–1244
**CPh** + **PS-FCS**	8.1 ^1^	4.2	42	10.4	17.2	1197
	6.1–14.9 ^2^	2.4–5.8	30–54	10.0–10.8	16.1–17.7	1149–1261
**CPh**	1.8 ^1^	0.4	12	2.5	4.2	645
	0.3–3.4 ^2^	0.1–0.9	11–25	1.5–9.4	3.0–15.9	203–1084
Control	16.2 ^1^	6.0	37	9.5	16.6	823
	13.9–16.9 ^2^	5.5–6.9	34–44	9.0–9.9	16.0–17.2	800–845

^1^ Median, ^2^ Minimum–maximum.

## Supplementary Materials

The following are available online at www.mdpi.com/1660-3397/15/10/301/s1, Table S1: Comparative analysis of hematological parameters in groups of mice after treatment of **CPh** with **r G-CSF**, **PS-Fuc**, **PS-FCS**.
