# Supplementary material for: Fucoidan and Fucosylated Chondroitin Sulfate Stimulate Hematopoiesis in Cyclophosphamide-Induced Mice
Source: Mar Drugs. 2017 Sep 30;15(10):301. doi: 10.3390/md15100301 (PMC5666409; doi:10.3390/md15100301)
Supplement: Supplementary file 1 [file marinedrugs-15-00301-s001.pdf]

Table S1. Comparative analysis of hematological parameters in groups of mice after treatment of CPh with G-CSF, **PS-Fuc**, **PS-FCS**

Parameters		**Groups**				
		CPh+r G-CSF	CPh+PS-Fuc	CPh+PS-FCS	CPh	Control
**WBCs**	p, vs CPh	0.008*	0.001*	0.041*	-	0.001*
	p, vs r G-CSF	-	0.345	0.741	0.008*	0.007*
	p, vs Control	0.007	0.001*	0.085	0.000	-
**Neutrophils, cells**	p, vs CPh	0.010*	0.004*	0.010*	-	0.001*
	p, vs r G-CSF	-	0.301	0.515	0.010*	0.418
	p, vs Control	0.418	0.014*	0.087	0.000	-
**Neutrophils, %**	p, vs CPh	0.068	0.068	0.067	-	0.068
	p, vs r G-CSF	-	0.698	1.000	0.069	0.171
	p, vs Control	0.171	0.648	1.000	0.068	-
**RBC**	p, vs CPh	0.072	0.052	0.073	-	0.105
	p, vs r G-CSF	-	0.536	0.724	0.072	0.226
	p, vs Control	0.226	0.001*	0.002*	0.105	-
**Platelets**	p, vs CPh	0.211	0.136	0.091	-	0.521
	p, vs r G-CSF	-	0.457	0.140	0.211	0.063
	p, vs Control	0.063	0.025*	0.001*	0.521	-
**Hemoglobin**	p, vs CPh	0.066	0.054	0.073	-	0.085
	p, vs r G-CSF	-	0.829	0.529	0.066	0.328
	p, vs Control	0.328	0.003*	0.154	0.085	-

$p<0.05$
